# Hypoxia During the Consolidation Phase of Distraction Osteogenesis Promotes Bone Regeneration

**DOI:** 10.3389/fphys.2022.804469

**Published:** 2022-02-22

**Authors:** Yanshi Liu, Jialin Liu, Feiyu Cai, Kai Liu, Xiaoxu Zhang, Aihemaitijiang Yusufu

**Affiliations:** ^1^Department of Trauma and Microreconstructive Surgery, The First Affiliated Hospital of Xinjiang Medical University, Ürümqi, China; ^2^Department of Prosthodontics, The First Affiliated Hospital of Xinjiang Medical University, Ürümqi, China; ^3^School of Public Health, Xinjiang Medical University, Ürümqi, China

**Keywords:** bone regeneration, consolidation, distraction osteogenesis, hypoxia, osteogenic-angiogenic coupling

## Abstract

**Background:**

Hypoxia is the critical driving force for angiogenesis and can trigger the osteogenic-angiogenic coupling followed by the enhancement of bone regeneration. While lots of studies showed that hypoxia administration can accelerate bone formation during distraction osteogenesis (DO), the therapeutic timing for the osteogenic purpose was concentrated on the distraction phase. The outcomes of hypoxia administration in the consolidation phase stay uncertain. The purpose of this study was to determine the osteogenic effectiveness of hypoxia therapy during the consolidation phase, if any, to enhance bone regeneration in a rat femoral DO model.

**Methods:**

A total of 42 adult male Sprague-Dawley rats underwent right femoral mid-diaphysis transverse osteotomy and were randomly divided into Control (NS administration, *n* = 21) and Group1 (deferoxamine therapy, *n* = 21) after distraction. During the consolidation phase, Group1 was treated with local deferoxamine (DFO) injection into the distraction zone, while the Control underwent the same dosage of NS. Animals were sacrificed after 2, 4, and 6 weeks of consolidation. The process of bone formation and remodeling was monitored by digital radiographs, and the regenerated bone was evaluated by micro-computed tomography (micro-CT), biomechanical test, and histological analysis. The serum content of hypoxia-inducible factor 1α (HIF-1α) and vascular endothelial growth factor (VEGF) were measured by enzyme linked immunosorbent assay (ELISA) for further analysis.

**Results:**

Bone regeneration was significantly enhanced after hypoxia therapy during the consolidation phase. The digital radiograph, micro-CT, and biomechanical evaluation showed better effects regarding volume, continuity, and mechanical properties of the regenerated bone in Group1. The histomorphological evaluation also revealed the hypoxia treatment contributed to accelerate bone formation and remodeling during DO. The higher positive expression of angiogenic and osteogenic markers were observed in Group1 after hypoxia administration according to the immunohistochemical analysis. The serum content of HIF-1α and VEGF was also increased after hypoxia therapy as evidenced from ELISA.

**Conclusion:**

Hypoxia administration during the consolidation phase of distraction osteogenesis has benefits in enhancing bone regeneration, including accelerates the bone formation and remodeling.

## Introduction

Distraction osteogenesis (DO), described by Ilizarov in the 1950s (Ilizarov, 1989a,b, 1990), has become a widely practiced technique in orthopedic and reconstructive surgery for limb lengthening, deformity correction, and bone defect treatment (Spiegl et al., 2013; Borzunov et al., 2015; Rohilla et al., 2016; El-Alfy et al., 2021; Liu et al., 2021). Compared with other alternative reconstructive methods, this technique provides advantages including avoidance of donor site morbidity and tissue concurrent generation using local endogenous substrate (Ai-Aql et al., 2008). Despite these clinical benefits, one of the limitations is the slow callus formation in the distraction gap. It needs a long duration for the regenerated bone to achieve final mineralization. The negatively social, psychological, and surgical complications may increase due to this long consolidation period with a bulky external frame (Paley, 1990). Various efforts have been developed to expedite bone regeneration during DO, including systemic or local addition of several pharmacological agents, osteogenic factors, or bone formation-inducing proteins (Alzahrani et al., 2018; Xu et al., 2018; Jia et al., 2019; Kumabe et al., 2020; Wang et al., 2021; Ye et al., 2021). However, most techniques are difficultly applied in clinical practice due to regulatory controls, uncertain therapeutic efficacy, and high cost (Alzahrani et al., 2018).

The regulated induction of new blood vessels contributes to bone formation and repair (Wang et al., 2007). Except for simply providing nutrients and oxygen, the intricate role of angiogenesis in bone formation plays a temporal and spatial interdependence with osteogenesis (Schipani et al., 2009). Tissue hypoxia is a critical driving force for angiogenesis and is under the control of the hypoxia-inducible factor (HIF) pathway (Maes et al., 2012). In addition, activation of the HIF pathway may stimulate osteogenic precursor recruitment and modulate subsequent function at the bone formation sites (Arnett, 2010). Considering the significant role of vascularization during bone regeneration, another way to improve the DO technique may be the augmentation of blood supply for the regenerated bone in the distraction gap.

Deferoxamine (DFO), an FDA approved iron chelator as hypoxia mimics, has been demonstrated to induce angiogenesis *via* the HIF pathway (Gleadle et al., 1995). Under normoxic conditions, the HIF-1α can rapidly degrade with the help of the necessary cofactor (Fe). DFO can indirectly stabilize the activity of HIF-1α *via* its chelation of iron (Cho et al., 2013), resulting in the accumulation of HIF-1α and activation of downstream angiogenic genes (Wan et al., 2008). Pre-clinical DFO administration for the enhancement of bone regeneration was firstly described by Wan et al. (2008). Subsequently, a series of researches conducted by Donneys et al. (2012, 2013) and Farberg et al. (2014) have determined the osteogenic effectiveness of local DFO injection into the distraction gap during DO, as well as some other similar studies (Farberg et al., 2012; Felice et al., 2013).

Although lots of previous studies have shown that tissue hypoxia has benefits in the acceleration of bone regeneration during DO, the timing of pharmacologic administration was all concentrated on the distraction phase. The outcomes of hypoxia therapy in the consolidation phase stay uncertain. We posited that hypoxia administration in the consolidation period might optimize bone formation during DO, and Buchman himself also said “there could also be a potential benefit for the use of DFO during the consolidation period” (Farberg et al., 2014). The purpose of this study was to determine the osteogenic effectiveness of hypoxia administration during the consolidation phase, if any, to enhance bone regeneration in a rat femoral DO model.

## Materials and Methods

### Animals

Adult male Sprague-Dawley rats weighing approximately 420 g were provided by the Experimental Animal Centre of Xinjiang Medical University [license No. SYXK (Xin) 2018-0003]. Rats were maintained in a pathogen-free environment and housed in a light/dark and temperature-controlled room. During a 7-day acclimation period before surgery, rats had free access to standard laboratory chow and sterile water. All procedures that involved animals were performed according to the Guide for the Care and Use of Laboratory Animals of The First Affiliated Hospital of Xinjiang Medical University. Ethics approval was obtained from the Animal Ethics Committee of First Affiliated Hospital of Xinjiang Medical University (No. IACUC-20200318-82).

### Surgical Procedures

**FIGURE 1:**
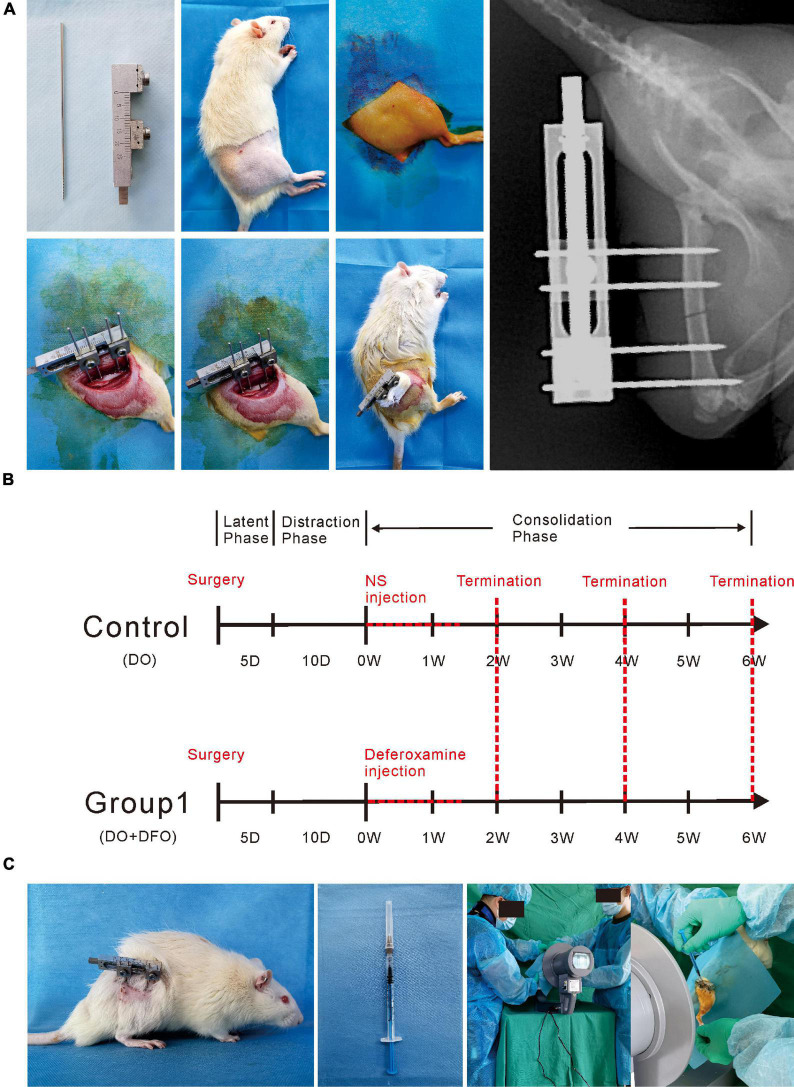
Methodologies of the present study. **(A)** The surgical procedures for the right femur model of distraction osteogenesis. **(B)** Rats were divided into two groups according to different interventions. DFO injection or NS administration as control was started from postoperative days 16 to 26. **(C)** Under image intensifier control, the solution was locally injected into the distraction gap every other day during the consolidation phase.

A total of 42 rats were used in the present study. All surgical procedures were performed by the same skilled team. Rats were anesthetized with 2% pentobarbital sodium (3 mg/100 g). A preoperative dose of benzylpenicillin was administered for infection prophylaxis. Under sterile conditions, a custom monolateral distraction external fixator (Designed and manufactured by the School of Mechanical Engineering, Xinjiang University) was installed on the right femur using four stainless steel self-tapping screws followed by a mid-diaphysis transverse osteotomy as previous described (Xu et al., 2017; Figure 1A).

### Postoperative Procedures and Hypoxia Administration Protocol

Pin sites care was performed using antibiotic solution daily, and a daily intramuscular injection of benzylpenicillin was conducted during the postoperative 3 days in all experimental rats for infection prevention. Rats were housed one per cage and free to move, chow, and water.

**FIGURE 2:**
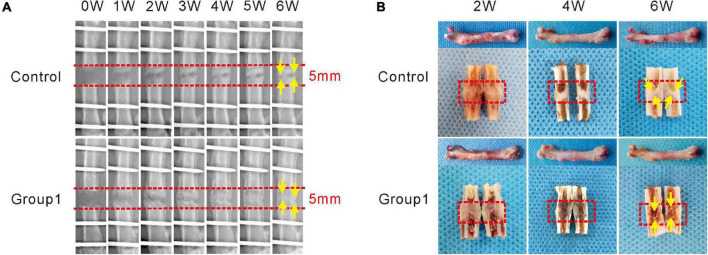
Hypoxia therapy during the consolidation phase accelerated bone regeneration in a rat femoral distraction osteogenesis model. The yellow arrows indicate bone union with primary recanalization of the medullary cavity was achieved in Group1 after 6 weeks of consolidation. **(A)** *X*-ray images of the distraction zone weekly until a 6-week consolidation duration was completed. **(B)** The general images of specimens after 2, 4, and 6 weeks of consolidation.

After a 5-day latency, the distraction was started at a rate of 0.25 mm/12 h for 10 days, producing a cumulative final gap distance of 5.0 mm followed by a 6-week consolidation duration. During the distraction period, no analgesic or sedation was required. Rats were divided into two groups randomly (Figure 2): control (*n* = 21), NS (300 μL) injection; Group1 (*n* = 21), DFO (200 μM in 300 μL NS) injection. During the injection, all rats underwent brief anesthesia using an isoflurane bell jar technique firstly. Starting from postoperative days 16 to 26, the solution was injected directly into the distraction zone every other day under the image intensifier control (Figures 1B,C). The dose of pharmacologic intervention was derived from previous studies (Street et al., 2002; Wan et al., 2008; Shen et al., 2009; Donneys et al., 2012, 2013; Farberg et al., 2012, 2014; Felice et al., 2013).

Rats in the two groups were sacrificed after 2, 4, and 6 weeks of consolidation (*n* = 6 per group at 2 and 4 weeks, *n* = 9 per group at 6 weeks). Bilateral femora without soft tissues were harvested for further analysis.

### Digital Radiographic Evaluation

After brief anesthesia by isoflurane, all the experimental rats underwent anteroposterior (AP) X-ray examination of the distraction zone weekly until sacrifice. All radiographs were conducted by the same digital X-ray apparatus (HF400VA, MIKASA X-RAY Co., Ltd., Tokyo, Japan) and condition (44 kV, 4.5 mAs).

### Micro-Computed Tomography Evaluation

After 6 weeks of consolidation, micro-CT imaging (Al + Cu filter, voxel size 18 μm, 65 kV, 385 μA for 340 ms; BRUKER Micro-CT SkyScan 1176, Bruker Physik-AG, Rheinstetten, Germany) was performed to evaluate the regenerated bone in the distraction zone (*n* = 3 per group). The Skyscan NRecon software was used for model reconstruction, and the three-dimensional (3D) algorithms in Skyscan CTAn software were used for the analysis according to the manufacturer’s instructions. Based on the previous study (Perrien et al., 2012), the distraction region surrounded by the outlined periosteum from the proximal to distal ends was defined as the region of interest (ROI). Tissues within the ROI were selected for the measurement of bone mineral density (BMD) and bone volume/total tissue volume (BV/TV).

### Biomechanical Test

After 6 weeks of consolidation, a three-point bending test was performed within 24 h at room temperature to evaluate the mechanical properties of samples (*n* = 3 per group). All external fixators, screws, and surrounding soft tissues were removed before the test. The unoperated femurs were used as controls. The long axis of the femur was aligned perpendicular to the blade with the span set as 18 mm, and then the distraction zone was constantly loaded in the AP direction at a loading rate of 0.5 mm/min until failure using a three-point bending apparatus (RGM-3005T, Shenzhen Reger Instrument Co., Ltd., China). Ultimate load, modulus of elasticity (E-modulus), energy to failure, and stiffness were recorded and calculated by the built-in software (REGER, Shenzhen Reger Instrument Co., Ltd., China), and they all were normalized to the contralateral femur.

### Histomorphological and Immunohistochemical Analysis

All samples were fixed in 10% formalin buffer for 48 h and then transferred to 75% ethanol for further analysis. At each time point, three specimens from each group were randomly selected for gradient alcohol dehydration and xylene defatting, and then embedded in methyl methacrylate. Sections 10 μm thick were cut using a hard tissue microtome (HistoCore AUTOCUT, Leica, Wetzlar, Germany). The sections underwent Von Kossa, Masson Trichrome, Goldner Trichrome, and Safranin O staining for static histomorphological observation. Under a magnification of 15×, sections (*n* = 3 per group) at each time point were semi-quantitatively analyzed by Image Pro Plus 6.0 software for the ratio of regenerated bone in the distraction zone.

After radiological evaluation, the other three specimens per group at each time point were decalcified in 10% ethylenediaminetetraacetic acid solution for 4 weeks, and then sample dehydration, transparency, and paraffin embedding were successively performed. Sections (5 μm) were cut using a microtome (RM2135, Leica, Wetzlar, Germany) for immunohistochemistry staining. After deparaffinization in xylene and rehydration in a graded series of alcohol, immunohistochemistry staining was performed following a standard protocol. Sections were incubated with primary antibodies anti-hypoxia-inducible factor 1α (anti-HIF-1α) (ab216842, Abcam, Cambridge, United Kingdom), anti-vascular endothelial growth factor (anti-VEGF) (sc7269, Santa Cruz, CA, United States), anti-runt-related transcription factor 2 (anti-RUNX2) (sc390351, Santa Cruz, CA, United States), anti-osterix (anti-Osx) (ab209484, Abcam, Cambridge, United Kingdom), anti-osteocalcin (anti-OCN) (23418-1-AP, Proteintech, Wuhan, China), anti-osteopontin (anti-OPN) (22952-1-AP, Proteintech, Wuhan, China) overnight at 4°C, and all dilution was 1:100 except for anti-Osx (1:400). After incubation in secondary antibody at 37°C for 1 h, a horseradish peroxidase-streptavidin system (ZLI-9019, ZSGB-BIO, Beijing, China) was used for signal detection followed by counterstaining using hematoxylin. Under a magnification of 200×, three fields in the distraction zone of each section were randomly selected for analysis (DP26, OLYMPUS, Japan). The positively stained area or cells were semi-quantitatively analyzed by Image Pro Plus 6.0 software.

### Blood Collection and Enzyme Linked Immunosorbent Assay Analysis

At each time point, blood samples (2 ml) were collected by cardiac puncture immediately (*n* = 6 per group). Serum was obtained *via* centrifugation at 1,800 rpm for 15 min at room temperature. Elisa kits were used for the evaluation of the angiogenic markers HIF-1α (JL20959, Shanghai Jianglai Industrial Limited By Share Ltd., Shanghai, China) and VEGF (JL21369, Shanghai Jianglai industrial Limited By Share Ltd., Shanghai, China) according to the manufacturer’s instructions.

### Statistical Analysis

The SPSS 22.0 (SPSS Inc., Chicago, IL, United States) was used for statistical analysis. All continuous variables were expressed as mean ± standard deviation (SD). The distribution of the data was evaluated by the Shapiro–Wilk test. The independent-samples *t*-test or Mann-Whitney *U* test was used to assess statistical differences. A statistically significant difference was set at *P* < 0.05. Graphs were derived from GraphPad Prism v.6.0 (GraphPad Inc., San Diego, CA, United States).

## Results

### Radiographic and Micro-Computed Tomography Evaluation

**FIGURE 3:**
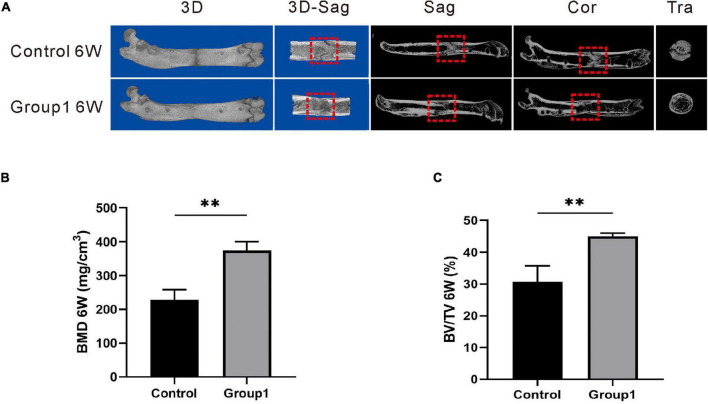
Results of micro-CT evaluation demonstrating promotion in regenerate quality after hypoxia therapy during the consolidation phase. **(A)** Representative 3D micro-CT images of the distraction zone at the termination of the 6-week consolidation. **(B,C)** Quantitative evaluation of BMD and BV/TV (*n* = 3 per group), manifesting both of the two values in Group1 were significantly higher than those in control (*p < 0.05, ^**^P < 0.01, independent *t*-test).

As shown in the representative images, there were some new callus in the distraction zone of the two groups after a 5-day distraction (0W), and the bone density became much higher at the termination of 6-week consolidation (Figure 2A). No significant differences in callus formation after 2 weeks between the two groups were observed. After the third week, bone formation was increased in Group1, and the difference between the two groups became more significant as time passed. After 4 weeks, there were abundant callus in the distraction zone of Group1, while an obvious gap was observed in Control. At the termination of the 6-week consolidation, bone union with primary recanalization of the medullary cavity was achieved in Group1, while there was a remaining gap in the distraction zone of Control. Similar results were observed in the gross inspection of the dissected specimens (Figure 2B) as well as the micro-CT examination (Figure 3A).

The representative images of micro-CT showed the marrow cavity of Group1 was almost completely remodeled after 6 weeks. However, there were abundant immature callus in the distraction zone of the Control, and the ongoing remodeling with a narrow gap was also observed (Figure 3A). In addition, the values of BMD (374.33 ± 25.72 mg/cm^3^) and BV/TV (45.00 ± 1.00%) in Group1 were significantly higher than those in control (227.67 ± 30.99 mg/cm^3^, 30.67 ± 5.03%) (*P* < 0.05) (Figures 3B,C). The results demonstrated the rate of bone remodeling was better in Group1, and the DFO therapy during the consolidation period contributed to enhancing bone formation and remodeling.

### Mechanical Properties Analysis

**FIGURE 4:**
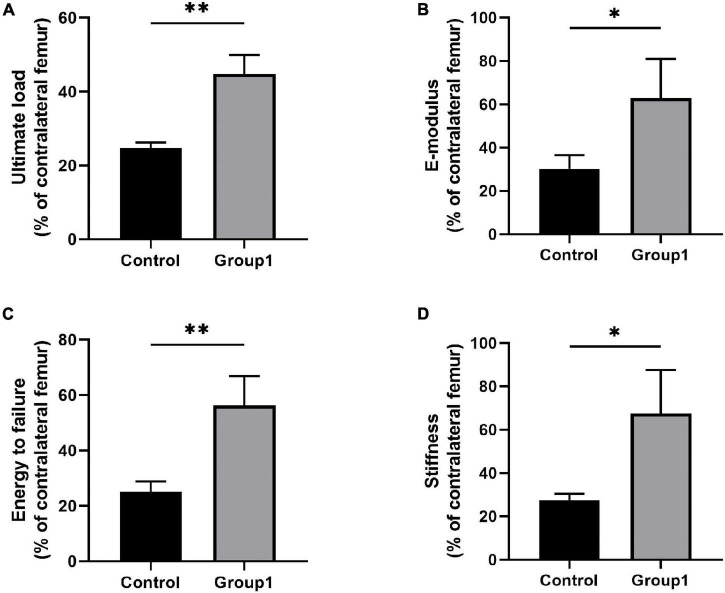
Results of mechanical properties and values were normalized to the contralateral femur (*n* = 3 per group) (*p < 0.05, ^**^P < 0.01, independent *t*-test).

A three-point bending test was carried out to evaluate the mechanical properties of samples after 6 weeks. Results showed there were significant improvement in ultimate load (44.79 ± 5.08%), E-modulus (62.89 ± 18.08%), energy to failure (56.27 ± 10.59%), and stiffness (67.56 ± 19.97%) of Group1 compared to Control (24.72 ± 1.47%, 30.23 ± 6.33%, 25.04 ± 3.78%, 27.41 ± 3.08%) (*P* < 0.05) (Figure 4).

### Histological Assessment

Von Kossa, Masson Trichrome, Goldner Trichrome, and Safranin O staining were used to assess the histomorphological characteristics of the undecalcified samples (2W, 4W, and 6W after consolidation). Von Kossa staining manifested an evident gap in the distraction zone of the two groups after 2 weeks of consolidation, but the ratio of regenerated bone was larger in Group1 (39.82 ± 2.38%) than that in Control (30.55 ± 3.74%) (*P* < 0.05). After 4 weeks, primarily continuous cortical bone was observed in the distraction zone of Group1, while an obvious gap still existed in Control. At the same time, there were more regenerated bones in Group1 (56.42 ± 3.74%) than that in Control (42.27 ± 1.09%) (*P* < 0.05). After 6 weeks, complete remodeling with recanalization of the medullary cavity was achieved in Group1, while abundant immature callus with a narrow gap and ongoing medullary cavity remodeling were observed in Control. Furthermore, the regenerated bone in the distraction zone of Group1 (26.54 ± 2.34%) was less than that in Control (48.05 ± 3.04%) (*P* < 0.05).

**FIGURE 5:**
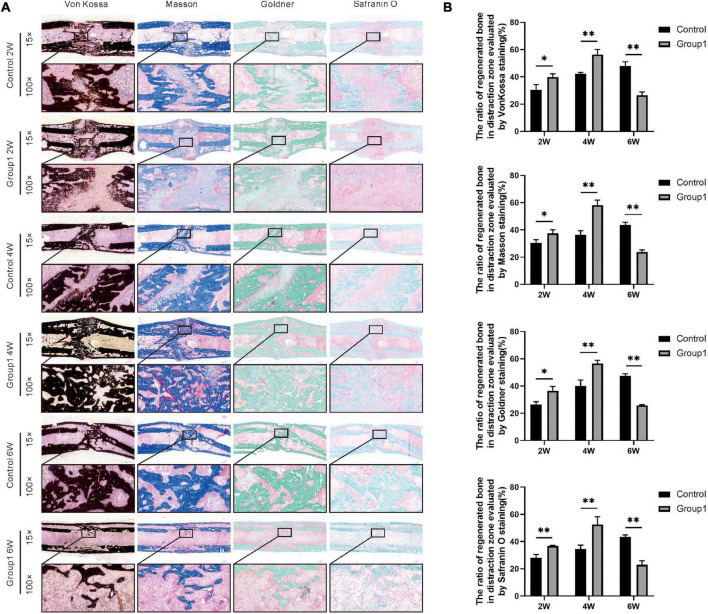
Histological analysis of bone regeneration during the consolidation phase. Von Kossa, Masson Trichrome, Goldner Trichrome, and Safranin O staining indicated the enhanced bone regeneration in the hypoxia therapy group (Group1). **(A)** Representative images of the histology sections. **(B)** Quantification of the ratio of regenerated bone in the distraction zone (*p < 0.05, ^**^P < 0.01, independent *t*-test).

Similar results were shown in Masson, Goldner, and Safranin O staining. There were more regenerated bone in Group1 (37.43 ± 2.59%, 36.42 ± 3.24%, 36.77 ± 0.39%) compared to Control (30.58 ± 2.26%, 26.41 ± 2.10%, 28.15 ± 1.91%) after 2 weeks (*P* < 0.05), the same as the termination of 4 weeks (58.24 ± 3.61%, 56.52 ± 2.38%, and 52.56 ± 4.62% in Group1, while 36.38 ± 3.01%, 26.41 ± 2.10%, 28.15 ± 1.91% in Control) (*P* < 0.05). Based on the aforementioned three stainings, the ratio of regenerated bone was also decreased in Group1 (23.74 ± 1.56%, 25.84 ± 0.43%, 22.94 ± 2.43%) after 6 weeks compared to Control (43.57 ± 2.08%, 47.55 ± 1.38%, 43.32 ± 1.29%) (*P* < 0.05). All the results demonstrated bone formation and remodeling were better in Group1. In addition, according to the Safranin O staining, the chondrocytes (cartilage) were observed evidently in the distraction zone of Control after 6 weeks, indicating the regenerated bone was not mineralized completely (Figure 5).

**FIGURE 6:**
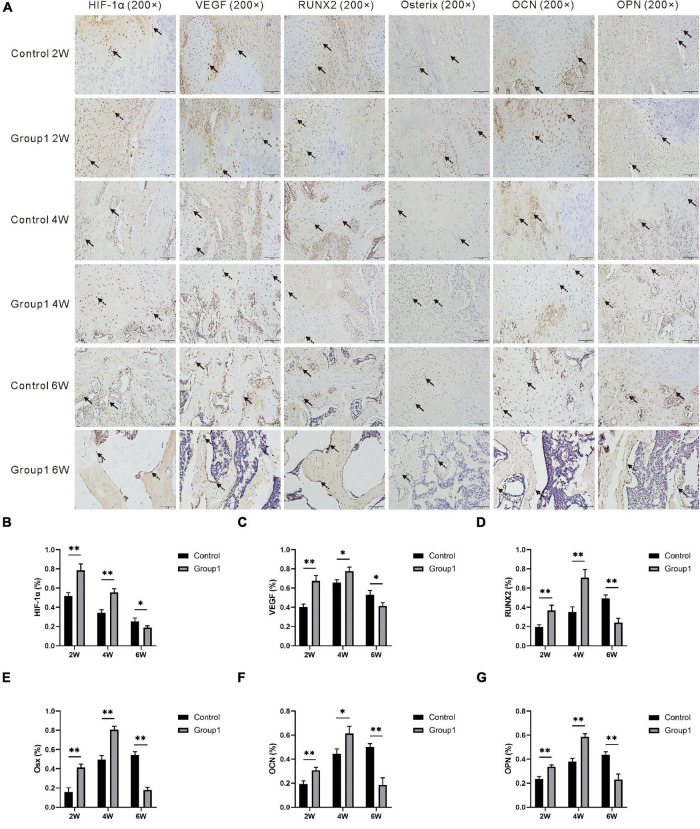
**(A)** Immunohistochemistry images of HIF-1α, VEGF, RUNX2, Osterix, OCN, and OPN in the two groups at the termination of 2-week, 4-week, and 6-week consolidation. Black arrows, positive cells in Control. Dotted arrows, positive cells in Group1. **(B–G)** The semiquantitative measurements (*n* = 3 per group) showed the 6 markers were highly expressed in Group1 compared to Control in the first 4 weeks consolidation duration. At the termination of the 6-week consolidation, HIF-1α and VEGF were expressed higher in Group1 than in Control, RUNX2 was expressed lower in Group1 than in Control, and the differences of the other three markers were not statistically significant (*p < 0.05, ^**^P < 0.01, independent *t*-test).

In the immunohistochemical analysis, the expression of HIF-1α, VEGF, RUNX2, Osterix, OCN, and OPN was increased in Group1 after the hypoxia administration at week 2 (*P* < 0.05). After 4 weeks, they were also higher expressed in Group1 than those in Control (*P* < 0.05). However, the aforementioned markers were lower expressed in Group1 compared to Control after 6 weeks (*P* < 0.05) (Figure 6).

### The Content of Hypoxia-Inducible Factor 1α and Vascular Endothelial Growth Factor in Serum

**FIGURE 7:**
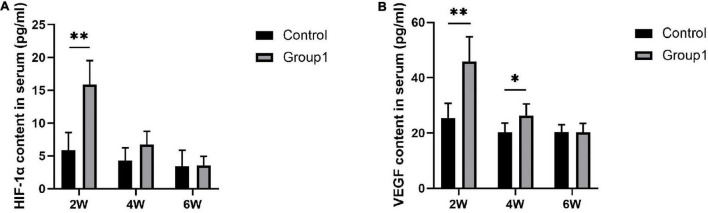
Serum content of HIF-1α and VEGF evaluated by ELISA (*n* = 3 per group). During the consolidation phase, the serum content of HIF-1α and VEGF were higher in Group1 compared to Control after 2 weeks. After 4 weeks, VEGF content was higher in Group1 than that in Control, while HIF-1α content was similar between the two groups. There was no statistical significance in the level of HIF-1α and VEGF between the two groups after 6 weeks (*p < 0.05, ^**^P < 0.01, independent *t*-test).

After 2 weeks of consolidation, the serum content of HIF-1α and VEGF showed the highest level in both the two groups, and they were higher expressed in Group1 (15.88 ± 3.64 pg/ml, 45.86 ± 8.97 pg/ml) compared to Control (5.87 ± 2.68 pg/ml, 25.47 ± 5.27 pg/ml) (*P* < 0.05). After 4 weeks, VEGF content was higher in Group1 (26.39 ± 4.14 pg/ml) than that in Control (20.22 ± 3.36 pg/ml) (*P* < 0.05), while no statistical difference of HIF-1α content was observed between the two groups (6.71 ± 2.04 pg/ml in Group1, 4.28 ± 1.94 pg/ml in Control). After 6 weeks, there was no statistical significance in HIF-1α (3.53 ± 1.41 pg/ml in Group1, 3.40 ± 2.45 pg/ml in Control) and VEGF (20.20 ± 3.26 pg/ml in Group1, 20.31 ± 2.67 pg/ml in Control) between the two groups (Figure 7).

## Discussion

The increased bone-forming activity that results from distraction is attributed to the stimulatory effect of tension on blood vessel formation and on the recruitment and proliferation of bone progenitor cells (Ai-Aql et al., 2008). Lots of published data have demonstrated that angiogenesis stimulated by the HIF pathway can increase bone regeneration during the osteogenic process (Ai-Aql et al., 2008; Schipani et al., 2009; Arnett, 2010; Maes et al., 2012). In the present study, we evaluated the therapeutic potential of hypoxia administration during the consolidation phase to enhance bone regeneration in a rat femoral distraction osteogenesis model, and results confirmed that there were beneficial effects on increasing osteogenesis and expediting bone consolidation.

Angiogenesis and vascular augmentation in the callus are critical mechanisms for successful bone regeneration during DO (Ilizarov, 1989a,b; Aronson, 1994). Although many studies attempted to enhance bone regeneration during DO using hypoxia mimics, to our knowledge, the therapeutic timing for the osteogenic purpose was all concentrated on the distraction phase. Here we specifically aimed to explore the effectiveness of different intervention timing, positing that hypoxia therapy in the consolidation period may also accelerate bone formation. The purpose of hypoxia administration in this study was to continuously enhance the natural vascular augmentation even further than the already hypervascular state triggered by the mechanical separation, and then expedited the bone regeneration indirectly.

In the present study, the digital radiographic results demonstrated that the bone quality in terms of volume and continuity of the callus was better in Group1 compared to Control after 6 weeks of consolidation. In addition, bone union with primary recanalization of the medullary cavity was macroscopically observed in Group1, similar results were shown in the micro-CT examination. The quantitative analysis regarding BMD, BV/TV, and mechanical properties of the distraction zone after 6 weeks also confirmed that hypoxia administration during the consolidation phase can obviously accelerate bone formation and remodeling. As for the histomorphological assessment, results manifested there was more regenerated bone in Group1 after 2 and 4 weeks, while the ratio of regenerated bone was decreased in Group1 after 6 weeks compared to Control. We considered that complete remodeling with recanalization of the medullary cavity was achieved in Group1 after 6 weeks, so there was less callus in the distraction zone. However, abundant immature callus with a narrow gap and ongoing medullary cavity remodeling were observed in Control after 6 weeks, therefore the ratio of regenerated bone was larger at that time. The above results manifested there was a better rate of bone formation and remodeling in Group1. These compelling findings foster tissue hypoxia during the consolidation phase as a potentially alternative means to promote bone regeneration in DO.

Brighton and Heppenstall (1971) demonstrated that pericellular reduction in oxygen tension is a hallmark of epiphyseal plates during bone development and is maintained within a fracture callus during bone repairment. The intimate relationship of the HIF pathway in the regulation of osteogenic-angiogenic coupling during bone regeneration has also been studied extensively (Wan et al., 2008; Schipani et al., 2009; Arnett, 2010; Maes et al., 2012). Wang et al. (2007) confirmed that the critical step of bone formation is VEGF-dependent blood vessel invasion into avascular cartilage. Wan et al. (2008) stated the HIF-1α and VEGF signaling triggered by tissue hypoxia would induce angiogenesis and osteogenesis. In this study, the immunohistochemical analysis manifested that HIF-1α was highly expressed in osteoblasts and chondrocytes after hypoxia therapy in Group1, and then decreased due to more trabecula was matured in the distraction zone. A similar phenomenon was also observed in the positive immunoreactivity trend of VEGF. However, the expression of HIF-1α and VEGF were lower expressed in Group1 after 6 weeks. Bone formation and remodeling were obviously accelerated in Group1 as evidence from the aforementioned evaluation, so there were few regenerated trabecula and active osteoblasts remained in the recanalized medullary cavity of Group1. We, therefore, speculated that fewer trabecula in Group1 produce less hypoxia relative factors and proteins. Additionally, the serum content of HIF-1α and VEGF showed the highest level in both the two groups after 2 weeks followed by decreasing gradually, and the values in Group1 were significantly higher than those in Control after 2 and 4 weeks, respectively. These findings suggested that local hypoxia resulted in HIF-1α and VEGF upregulation, and then stimulated the osteogenic cascade activity to improve mineralization.

During osteoblast differentiation and bone regeneration, RUNX2, Osx, OCN, and OPN are osteogenic markers (Baek et al., 2009; Shao et al., 2021), and they were gradually increased in the first 4 weeks of the mineralization period in this study. It was worth noting that the four indicators were higher expressed in Group1 than those in Control after hypoxia administration at week 2 and 4, while lower expressed in Group1 after 6 weeks. We also speculated that better remodeling resulted in few regenerated trabecula and active osteoblasts in Group1 followed by less production of osteogenic relative factors and proteins.

Despite our promising findings, there were several limitations in this study. First of all, the present study is a sort of preliminary investigation to pave a way for many future works aiming to make the DO process more efficient, a prudent attitude should be adopted regarding the superiority of intervention timing due to the absence of hypoxia therapy in the distraction phase, and future directions may consider the best timing through the comparison of hypoxia administration at different phases. In addition, different delivery methods, dosages, and timing need to be optimized for superior effectiveness. Finally, histologic and morphologic characteristics of the regenerated bone were used as the main principle for the effectiveness evaluation in this study, a subsequent study is also needed to detailly investigate the molecular mechanisms about the HIF pathway that underlie the observed effects.

In summary, our treatment protocol of hypoxia administration was aimed to promote bone regeneration in DO, and the results demonstrated that the bone regeneration was indirectly enhanced by the HIF pathway triggered by local tissue hypoxia during the consolidation phase. Future directions may consider the controlled local release to avoid the need for repeated injections. Furthermore, it is worth investigating the relationships between the different intervention timing, dose response, and consolidation rate for superior practicability.

## Conclusion

Our study demonstrates the osteogenic effectiveness of hypoxia therapy during the consolidation phase to enhance bone regeneration in a rat femoral distraction osteogenesis model, including accelerates bone formation and remodeling. Although the aforementioned limitations are needed to be resolved by further studies, the results suggest that hypoxia administration may become a potentially alternative treatment to improve bone regeneration during DO.

## Data Availability Statement

The original contributions presented in the study are included in the article/Supplementary Material, further inquiries can be directed to the corresponding author/s.

## Ethics Statement

The animal study was reviewed and approved by the Animal Ethics Committee of The First Affiliated Hospital of Xinjiang Medical University. Written informed consent was obtained from the owners for the participation of their animals in this study.

## Author Contributions

YL, JL, and AY: conceptualization. YL and JL: methodology. YL: software and writing original draft preparation. YL, JL, FC, and KL: investigation. YL, JL, and XZ: data analysis. JL, FC, KL, XZ, and AY: review and editing. AY: supervision. JL and AY: funding acquisition. All authors have read and agreed to the published version of the manuscript.

## Conflict of Interest

The authors declare that the research was conducted in the absence of any commercial or financial relationships that could be construed as a potential conflict of interest.

## Publisher’s Note

All claims expressed in this article are solely those of the authors and do not necessarily represent those of their affiliated organizations, or those of the publisher, the editors and the reviewers. Any product that may be evaluated in this article, or claim that may be made by its manufacturer, is not guaranteed or endorsed by the publisher.

## References

[B1] Ai-Aql Z. S. Alagl A. S. Graves D. T. Gerstenfeld L. C. Einhorn T. A. (2008). Molecular mechanisms controlling bone formation during fracture healing and distraction osteogenesis. *J. Dent Res.* 87 107–118. 10.1177/154405910808700215 18218835 PMC3109437

[B2] Alzahrani M. M. Anam E. AlQahtani S. M. Makhdom A. M. Hamdy R. C. (2018). Strategies of enhancing bone regenerate formation in distraction osteogenesis. *Connect Tissue Res.* 59 1–11. 10.1080/03008207.2017.1288725 28165797

[B3] Arnett T. R. (2010). Acidosis, hypoxia and bone. *Arch. Biochem. Biophys.* 503 103–109. 10.1016/j.abb.2010.07.021 20655868

[B4] Aronson J. (1994). Temporal and spatial increases in blood flow during distraction osteogenesis. *Clin. Orthop. Relat. Res.* 1994 124–131. 10.1097/00003086-199404000-00020 8156663

[B5] Baek W. Y. Lee M. A. Jung J. W. Kim S. Y. Akiyama H. de Crombrugghe B. (2009). Positive regulation of adult bone formation by osteoblast-specific transcription factor osterix. *J. Bone Miner. Res.* 24 1055–1065. 10.1359/jbmr.081248 19113927 PMC4020416

[B6] Borzunov D. Y. Balaev P. I. Subramanyam K. N. (2015). Reconstruction by bone transport after resection of benign tumors of tibia: a retrospective study of 38 patients. *Indian J. Orthop.* 49 516–522. 10.4103/0019-5413.164042 26538757 PMC4598542

[B7] Brighton C. T. Heppenstall R. B. (1971). Oxygen tension in zones of the epiphyseal plate, the metaphysis and diaphysis. An in vitro and in vivo study in rats and rabbits. *J. Bone Joint Surg. Am.* 53 719–728. 10.2106/00004623-197153040-00011 5580029

[B8] Cho E. A. Song H. K. Lee S. H. Chung B. H. Lim H. M. Lee M. K. (2013). Differential in vitro and cellular effects of iron chelators for hypoxia inducible factor hydroxylases. *J. Cell Biochem.* 114 864–873. 10.1002/jcb.24423 23097160

[B9] Donneys A. Deshpande S. S. Tchanque-Fossuo C. N. Johnson K. L. Blough J. T. Perosky J. E. (2013). Deferoxamine expedites consolidation during mandibular distraction osteogenesis. *Bone* 55 384–390. 10.1016/j.bone.2013.04.005 23598047 PMC4162399

[B10] Donneys A. Farberg A. S. Tchanque-Fossuo C. N. Deshpande S. S. Buchman S. R. (2012). Deferoxamine enhances the vascular response of bone regeneration in mandibular distraction osteogenesis. *Plast Reconstr. Surg.* 129 850–856. 10.1097/PRS.0b013e31824422f2 22456357 PMC4535714

[B11] El-Alfy B. S. Maaty M. Niazy T. (2021). Reconstruction of infected nonunion of the distal humerus by Ilizarov external fixator. *Injury* 52 1418–1422. 10.1016/j.injury.2020.10.073 33139035

[B12] Farberg A. S. Jing X. L. Monson L. A. Donneys A. Tchanque-Fossuo C. N. Deshpande S. S. (2012). Deferoxamine reverses radiation induced hypovascularity during bone regeneration and repair in the murine mandible. *Bone* 50 1184–1187. 10.1016/j.bone.2012.01.019 22314387 PMC3322244

[B13] Farberg A. S. Sarhaddi D. Donneys A. Deshpande S. S. Buchman S. R. (2014). Deferoxamine enhances bone regeneration in mandibular distraction osteogenesis. *Plast Reconstr. Surg.* 133 666–671. 10.1097/01.prs.0000438050.36881.a9 24572857 PMC4484577

[B14] Felice P. A. Ahsan S. Donneys A. Deshpande S. S. Nelson N. S. (2013). Deferoxamine administration delivers translational optimization of distraction osteogenesis in the irradiated mandible. *Plast Reconstr. Surg.* 132 542e–548e. 10.1097/PRS.0b013e31829fe548 24076701 PMC3787312

[B15] Gleadle J. M. Ebert B. L. Firth J. D. Ratcliffe P. J. (1995). Regulation of angiogenic growth factor expression by hypoxia, transition metals, and chelating agents. *Am. J. Physiol.* 268 C1362–C1368. 10.1152/ajpcell.1995.268.6.C1362 7541940

[B16] Ilizarov G. A. (1989a). The tension-stress effect on the genesis and growth of tissues. Part I. The influence of stability of fixation and soft-tissue preservation. *Clin. Orthop. Relat. Res.* 1989 249–281. 2910611

[B17] Ilizarov G. A. (1989b). The tension-stress effect on the genesis and growth of tissues: Part II. The influence of the rate and frequency of distraction. *Clin. Orthop. Relat. Res.* 1989 263–285. 10.1007/978-1-4471-5451-8_136 2912628

[B18] Ilizarov G. A. (1990). Clinical application of the tension-stress effect for limb lengthening. *Clin. Orthop. Relat. Res.* 1990 8–26. 10.1097/00003086-199001000-00003 2403497

[B19] Jia Y. Zhu Y. Qiu S. Xu J. Chai Y. (2019). Exosomes secreted by endothelial progenitor cells accelerate bone regeneration during distraction osteogenesis by stimulating angiogenesis. *Stem Cell Res. Ther.* 10:12. 10.1186/s13287-018-1115-7 30635031 PMC6329174

[B20] Kumabe Y. Fukui T. Takahara S. Kuroiwa Y. Arakura M. Oe K. (2020). Percutaneous CO2 treatment accelerates bone generation during distraction osteogenesis in rabbits. *Clin. Orthop. Relat. Res.* 478 1922–1935. 10.1097/CORR.0000000000001288 32732577 PMC7371043

[B21] Liu Y. Yushan M. Liu Z. Liu J. Ma C. Yusufu A. (2021). Treatment of diaphyseal forearm defects caused by infection using Ilizarov segmental bone transport technique. *BMC Musculoskelet. Disord* 22:36. 10.1186/s12891-020-03896-w 33413259 PMC7789280

[B22] Maes C. Carmeliet G. Schipani E. (2012). Hypoxia-driven pathways in bone development, regeneration and disease. *Nat. Rev. Rheumatol.* 8 358–366. 10.1038/nrrheum.2012.36 22450551

[B23] Paley D. (1990). Problems, obstacles, and complications of limb lengthening by the Ilizarov technique. *Clin. Orthop. Relat. Res.* 1990 81–104. 10.1007/BF00636173 2403498

[B24] Perrien D. S. Nicks K. M. Liu L. Akel N. S. Bacon A. W. Skinner R. A. (2012). Inhibin a enhances bone formation during distraction osteogenesis. *J. Orthop. Res.* 30 288–295. 10.1002/jor.21501 21809377 PMC3737578

[B25] Rohilla R. Siwach K. Devgan A. Singh R. Wadhwani J. Ahmed N. (2016). Outcome of distraction osteogenesis by ring fixator in infected, large bone defects of tibia. *J. Clin. Orthop. Trauma* 7 201–209. 10.1016/j.jcot.2016.02.016 28053385 PMC5197053

[B26] Schipani E. Maes C. Carmeliet G. Semenza G. L. (2009). Regulation of osteogenesis-angiogenesis coupling by HIFs and VEGF. *J. Bone Miner. Res.* 24 1347–1353. 10.1359/jbmr.090602 19558314 PMC3276346

[B27] Shao H. Wu R. Cao L. Gu H. Chai F. (2021). Trelagliptin stimulates osteoblastic differentiation by increasing runt-related transcription factor 2 (RUNX2): A therapeutic implication in osteoporosis. *Bioengineered* 12 960–968. 10.1080/21655979.2021.1900633 33734011 PMC8291811

[B28] Shen X. Wan C. Ramaswamy G. Mavalli M. Wang Y. Duvall C. L. (2009). Prolyl hydroxylase inhibitors increase neoangiogenesis and callus formation following femur fracture in mice. *J. Orthop. Res.* 27 1298–1305. 10.1002/jor.20886 19338032 PMC3767389

[B29] Spiegl U. Patzold R. Friederichs J. Hungerer S. Militz M. Bühren V. (2013). Clinical course, complication rate and outcome of segmental resection and distraction osteogenesis after chronic tibial osteitis. *Injury* 44 1049–1056. 10.1016/j.injury.2013.05.003 23747125

[B30] Street J. Bao M. DeGuzman L. Bunting S. Peale F. J. (2002). Vascular endothelial growth factor stimulates bone repair by promoting angiogenesis and bone turnover. *Proc Natl Acad Sci U S a* 99 9656–9661. 10.1073/pnas.152324099 12118119 PMC124965

[B31] Wan C. Gilbert S. R. Wang Y. Cao X. Shen X. Ramaswamy G. (2008). Activation of the hypoxia-inducible factor-1α pathway accelerates bone regeneration. *Proc. Natl. Acad. Sci. U.S.A.* 105, 686–691. 10.1073/pnas.0708474105 18184809 PMC2206597

[B32] Wang F. Qian H. Kong L. Wang W. Wang X. Xu Z. (2021). Accelerated bone regeneration by astragaloside IV through stimulating the coupling of osteogenesis and angiogenesis. *Int. J. Biol. Sci.* 17, 1821–1836. 10.7150/ijbs.57681 33994865 PMC8120474

[B33] Wang Y. Wan C. Gilbert S. R. Clemens T. L. (2007). Oxygen sensing and osteogenesis. *Ann. N. Y. Acad. Sci.* 1117, 1–11. 10.1196/annals.1402.049 18056033

[B34] Xu J. Sun Y. Wu T. Liu Y. Shi L. Zhang J. (2018). Enhancement of bone regeneration with the accordion technique via HIF-1α/VEGF activation in a rat distraction osteogenesis model. *J. Tissue Eng. Regen. Med.* 12, e1268–e1276. 10.1002/term.2534 28763580

[B35] Xu J. Wu T. Sun Y. Wang B. Zhang J. Yuk-Wai Lee W. (2017). Staphylococcal enterotoxin C2 expedites bone consolidation in distraction osteogenesis. *J. Orthop. Res.* 35, 1215–1225. 10.1002/jor.23372 27431811

[B36] Ye L. Xu J. Mi J. He X. Pan Q. Zheng L. (2021). Biodegradable magnesium combined with distraction osteogenesis synergistically stimulates bone tissue regeneration via CGRP-FAK-VEGF signaling axis. *Biomaterials* 275:120984. 10.1016/j.biomaterials.2021.120984 34186235

