# Supplementary figures and images for: Hypoxia During the Consolidation Phase of Distraction Osteogenesis Promotes Bone Regeneration
Source: Front Physiol. 2022 Feb 22;13:804469. doi: 10.3389/fphys.2022.804469 (PMC8905603; doi:10.3389/fphys.2022.804469)

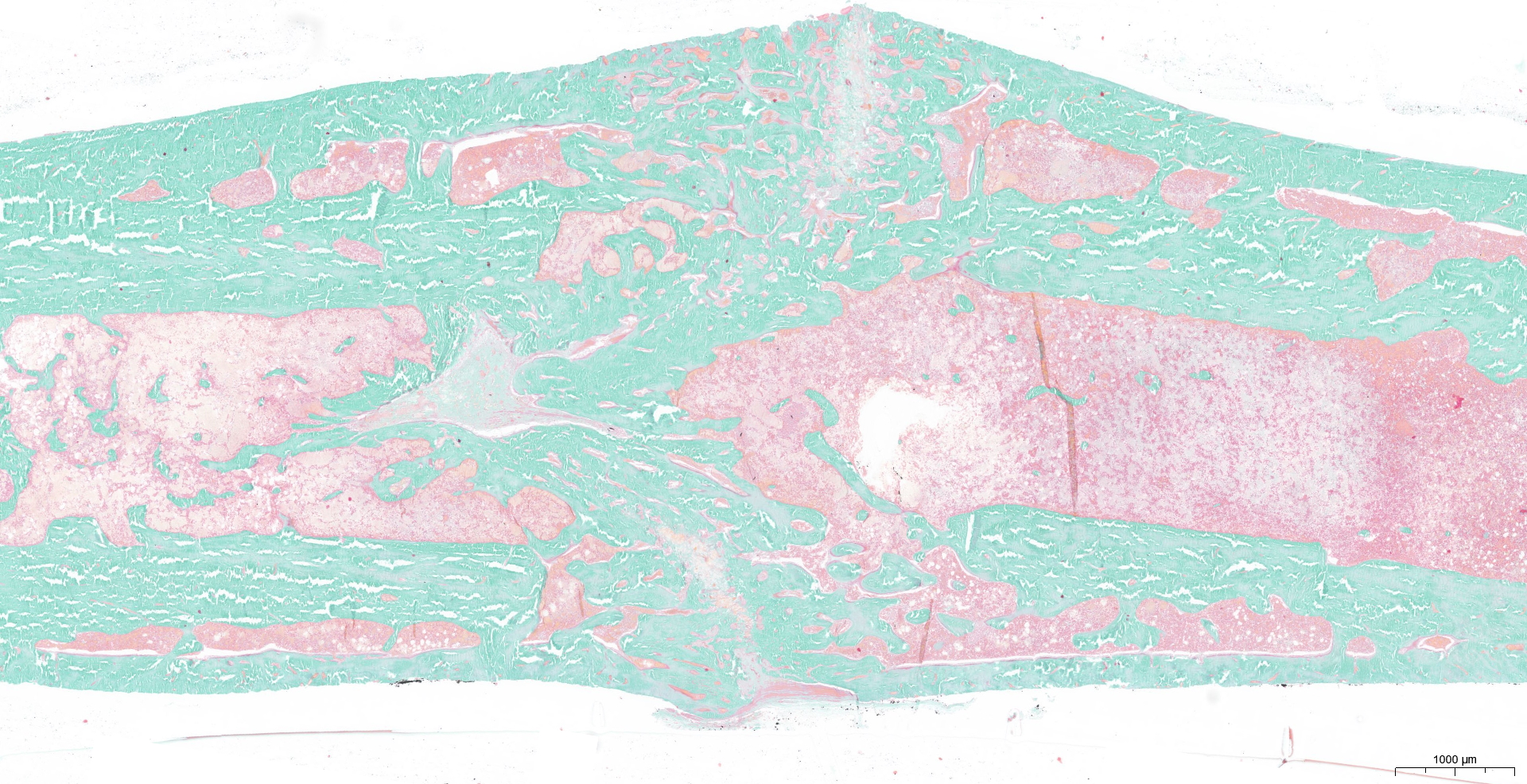

Supplement: Supplementary file 1 [file Presentation_1.zip › (Fro revision 3)Images of histomorphological analysis/Group1-4W/K18-4W Goldner_1.5x.jpg]

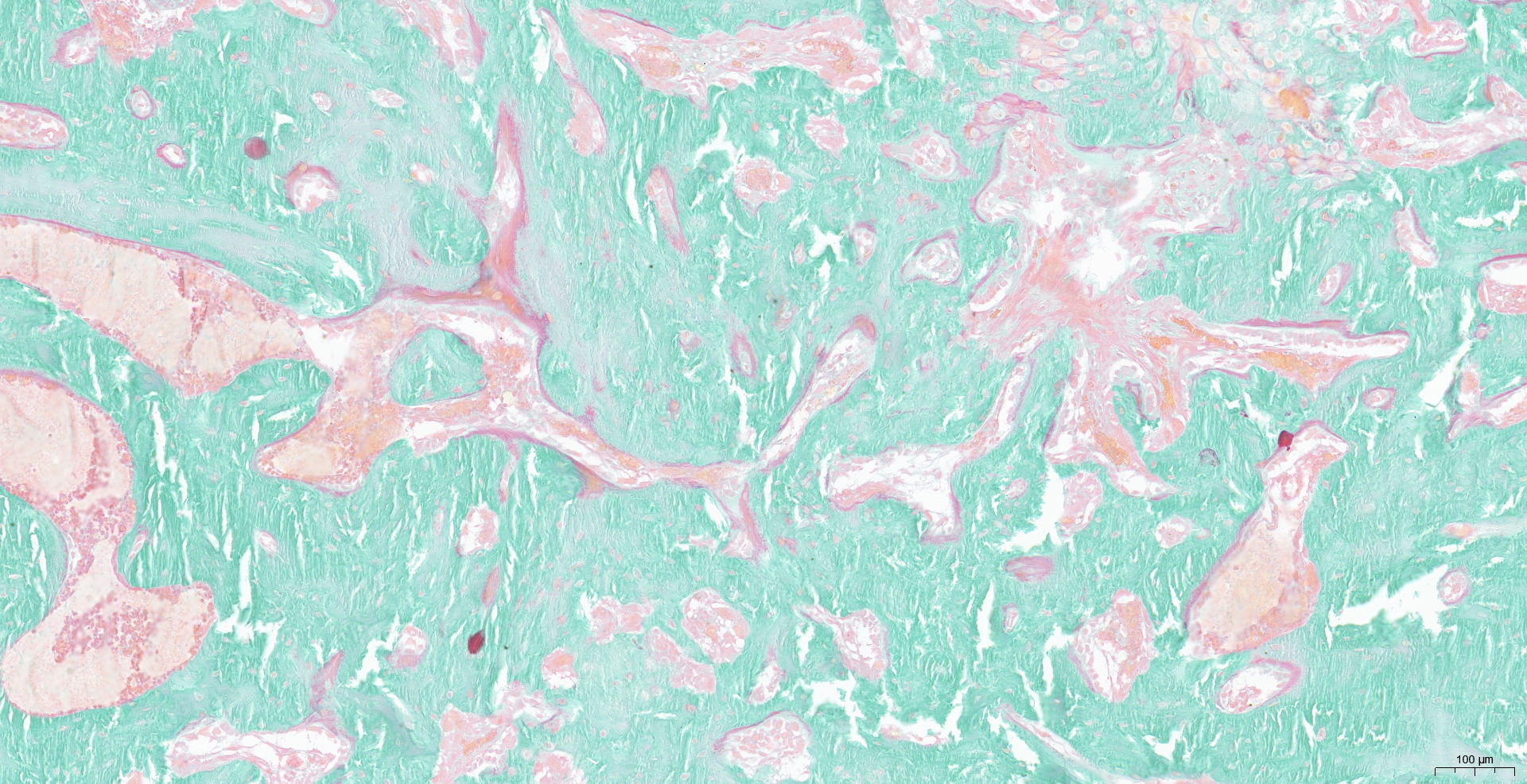

Supplement: Supplementary file 1 [file Presentation_1.zip › (Fro revision 3)Images of histomorphological analysis/Group1-4W/K18-4W Goldner_10.0x.jpg]

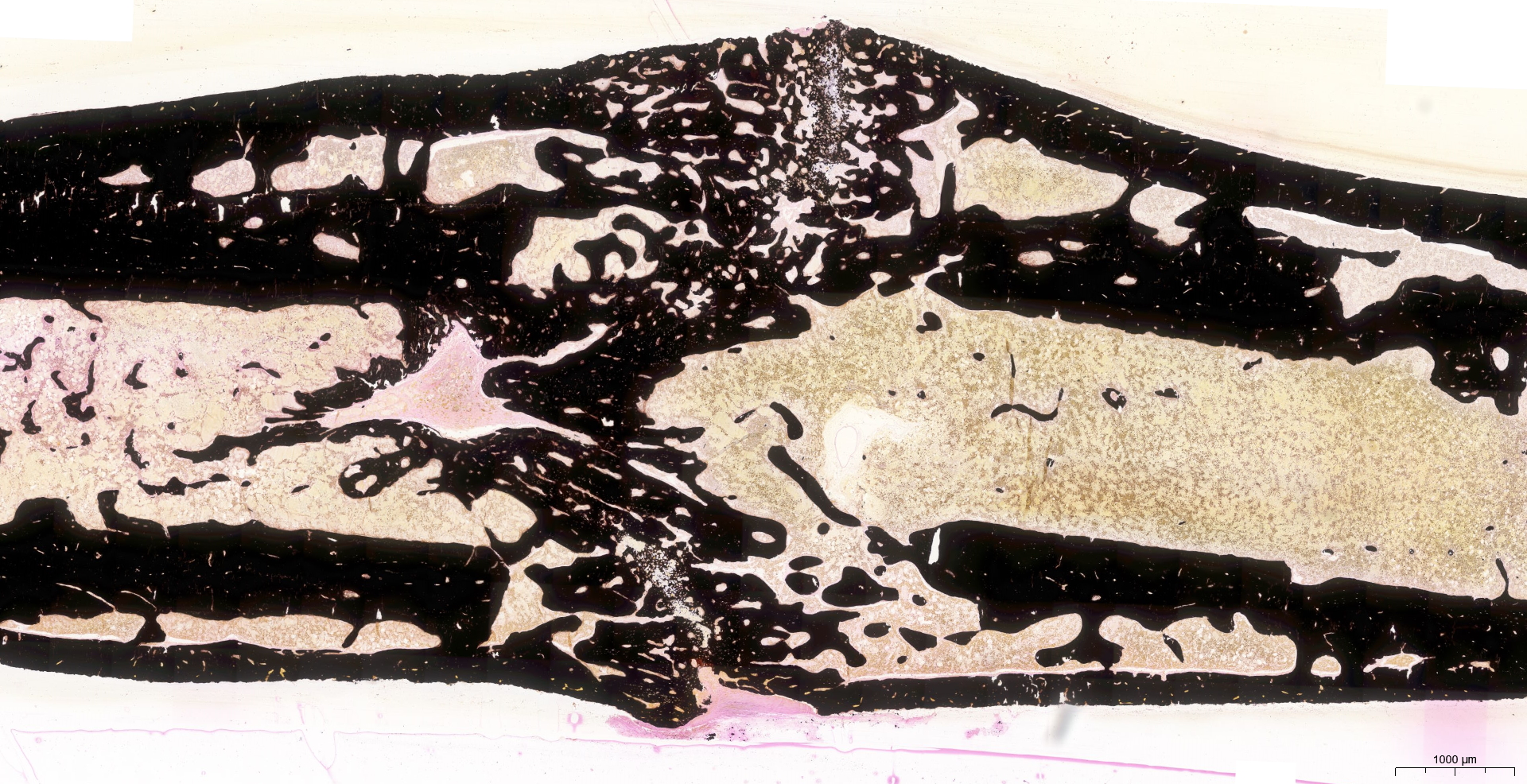

Supplement: Supplementary file 1 [file Presentation_1.zip › (Fro revision 3)Images of histomorphological analysis/Group1-4W/K18-4W VonKossa_1.5x.jpg]

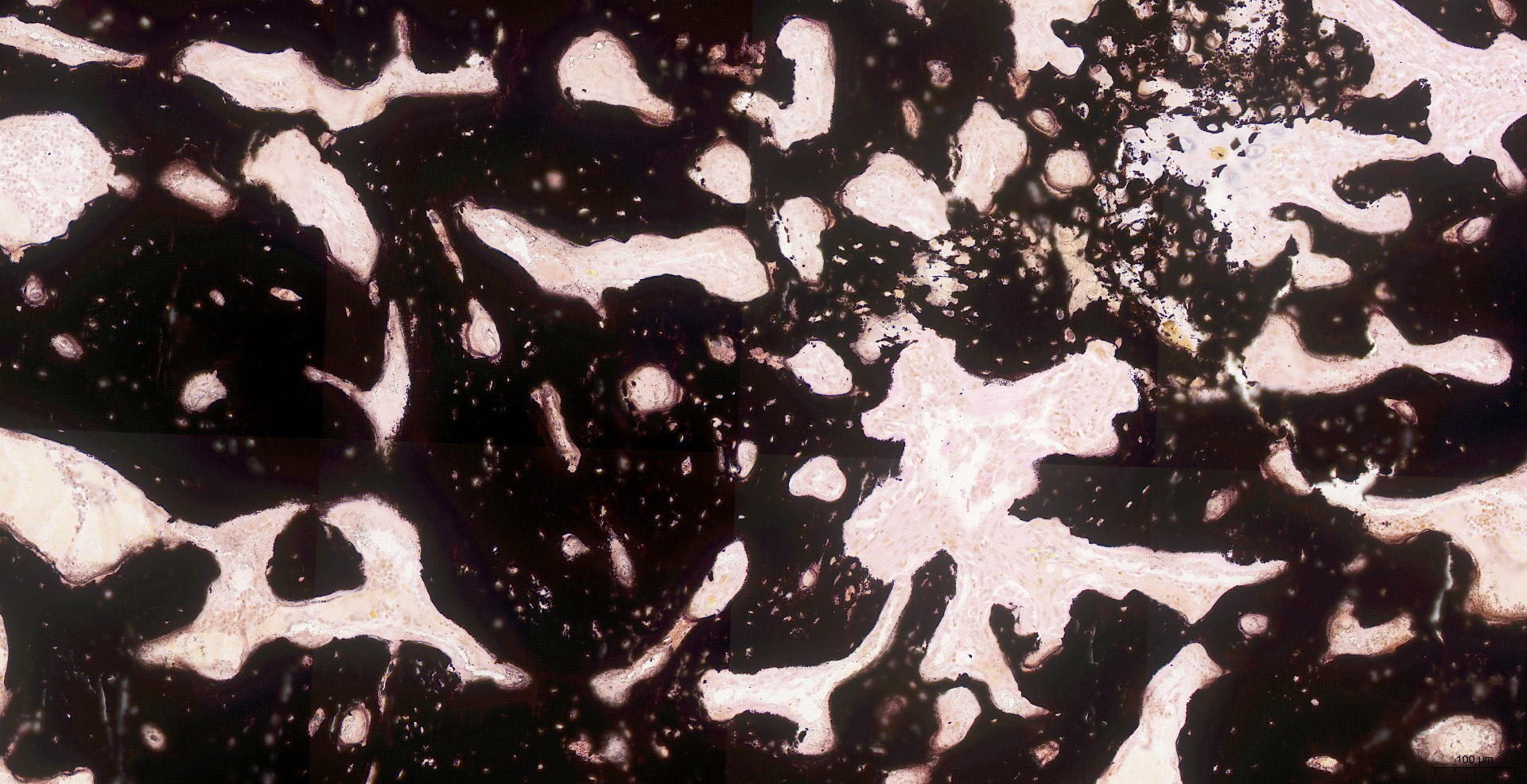

Supplement: Supplementary file 1 [file Presentation_1.zip › (Fro revision 3)Images of histomorphological analysis/Group1-4W/K18-4W VonKossa_10.0x.jpg]

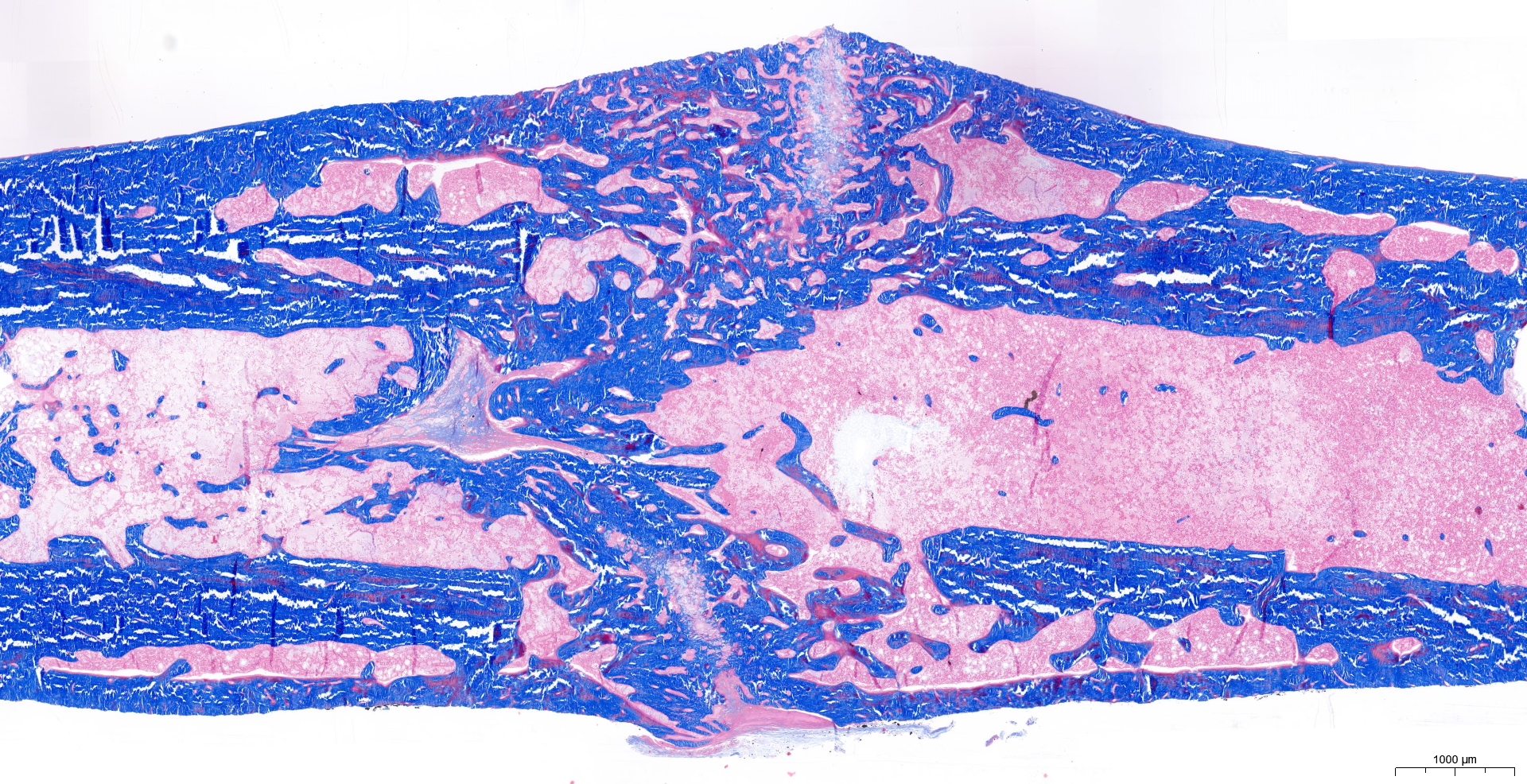

Supplement: Supplementary file 1 [file Presentation_1.zip › (Fro revision 3)Images of histomorphological analysis/Group1-4W/K18-4W masson_1.5x.jpg]

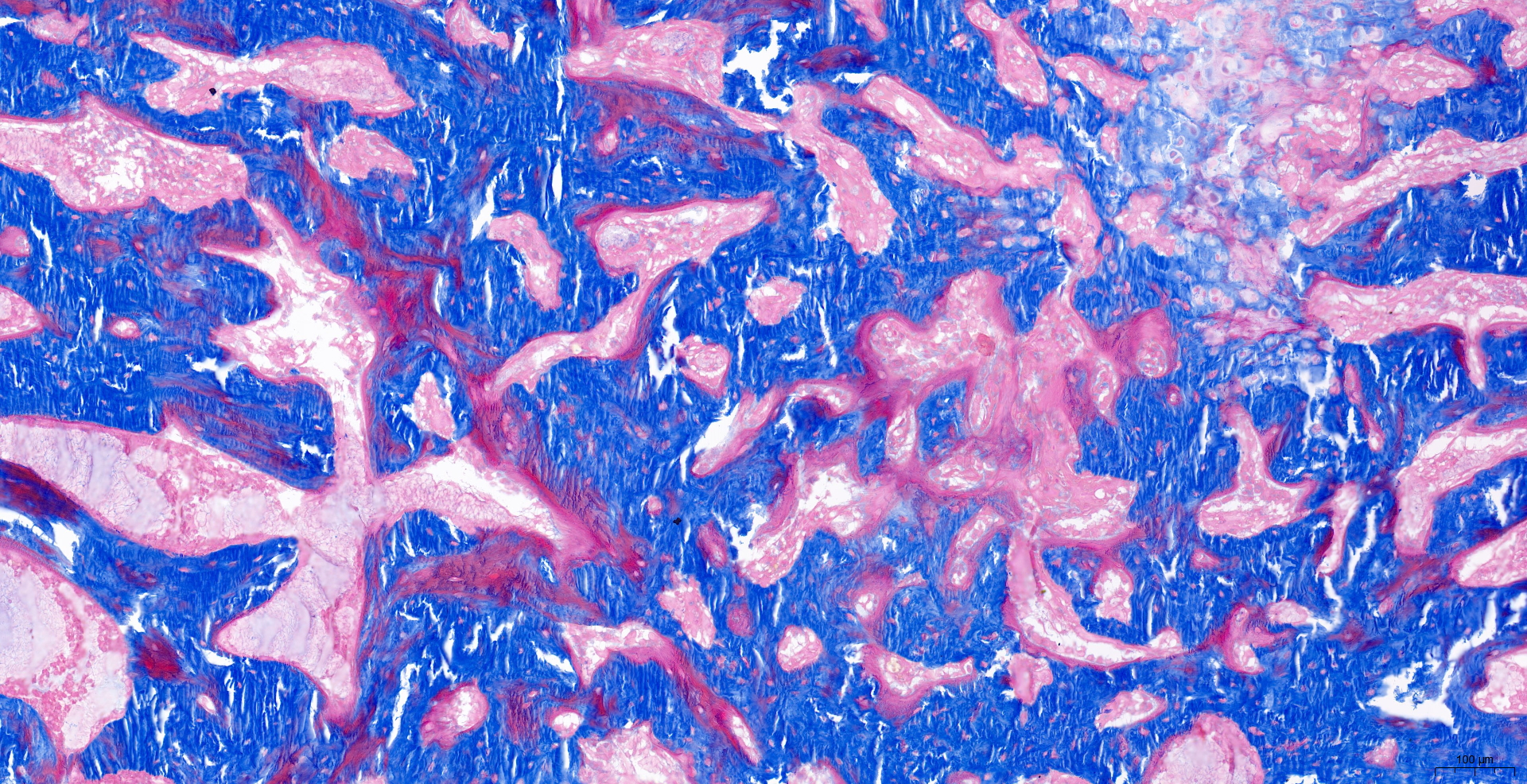

Supplement: Supplementary file 1 [file Presentation_1.zip › (Fro revision 3)Images of histomorphological analysis/Group1-4W/K18-4W masson_10.0x.jpg]

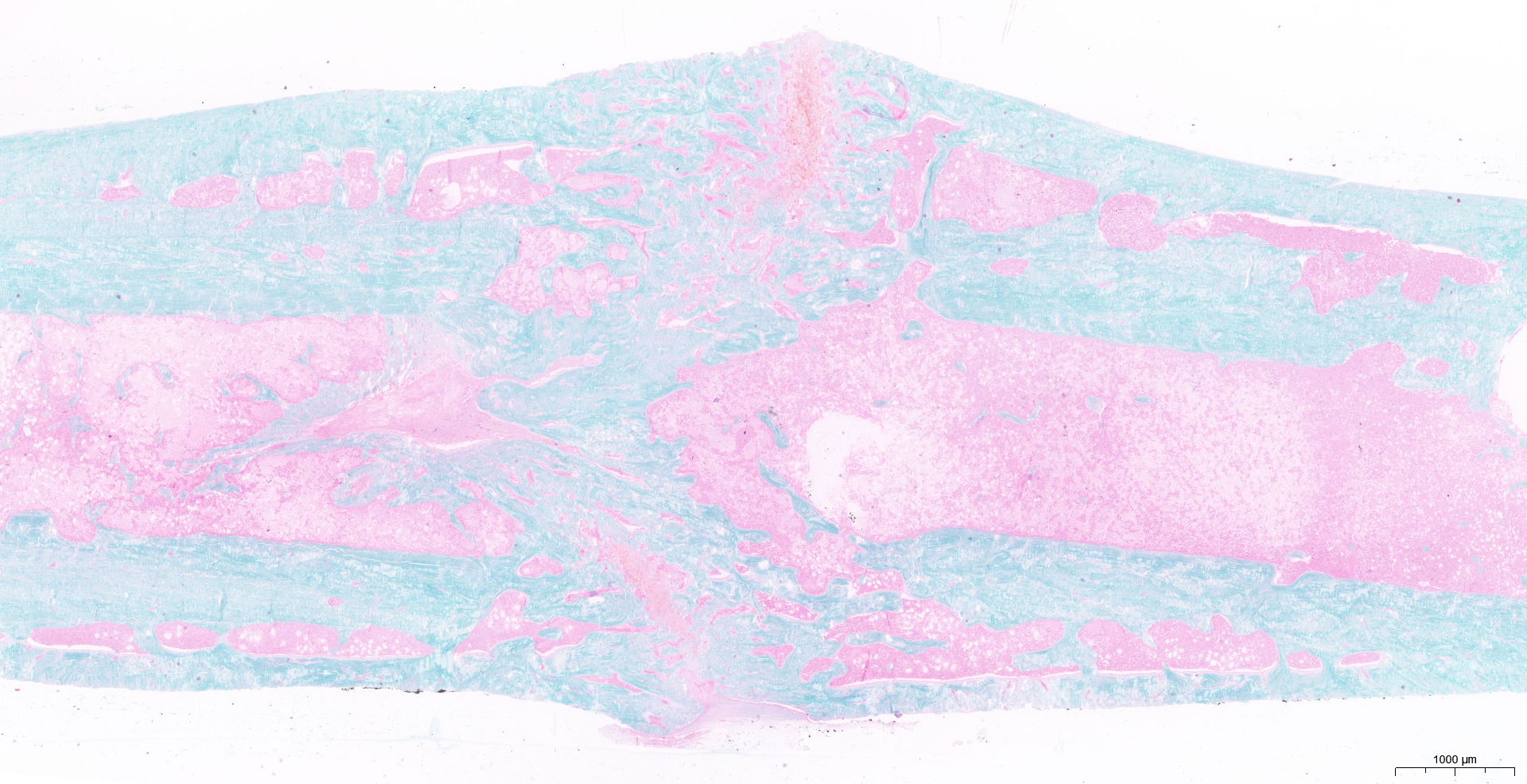

Supplement: Supplementary file 1 [file Presentation_1.zip › (Fro revision 3)Images of histomorphological analysis/Group1-4W/K18-4W ╖1⁄4╣╠_1.5x1.jpg]

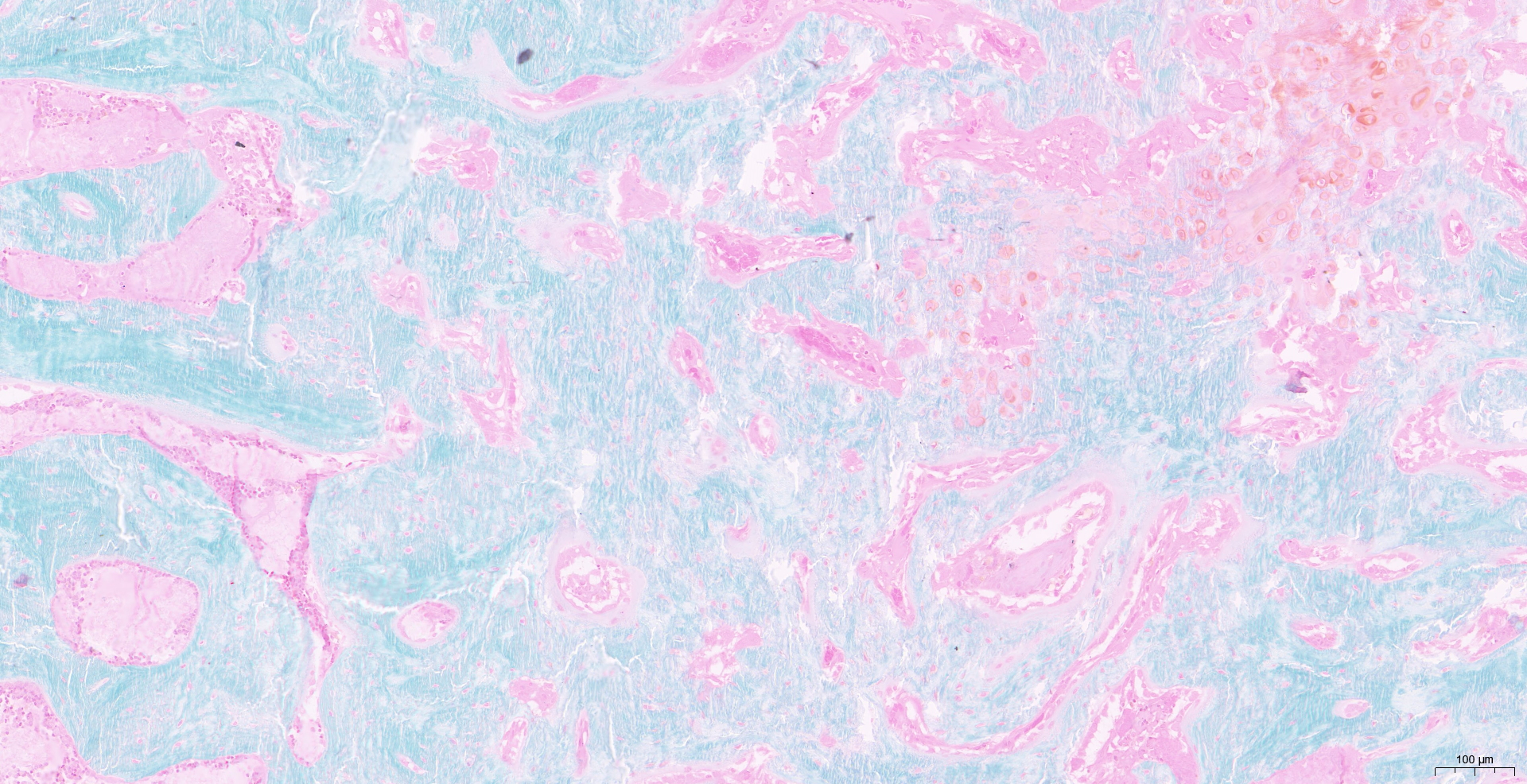

Supplement: Supplementary file 1 [file Presentation_1.zip › (Fro revision 3)Images of histomorphological analysis/Group1-4W/K18-4W ╖1⁄4╣╠_10.0x.jpg]
