# Supplementary material for: Hypoxia During the Consolidation Phase of Distraction Osteogenesis Promotes Bone Regeneration
Source: Front Physiol. 2022 Feb 22;13:804469. doi: 10.3389/fphys.2022.804469 (PMC8905603; doi:10.3389/fphys.2022.804469)
Supplement: Supplementary file 7 [file Table_6.docx]

https://www.jianguoyun.com/p/DZv76EoQwd78CRjc_ZsE

https://www.jianguoyun.com/p/DY45Ht8Qwd78CRjW4aME
